# Herpesviruses possess conserved proteins for interaction with Nedd4 family ubiquitin E3 ligases

**DOI:** 10.1038/s41598-018-22682-2

**Published:** 2018-03-13

**Authors:** Tetsuo Koshizuka, Takahiro Kobayashi, Ken Ishioka, Tatsuo Suzutani

**Affiliations:** Department of Microbiology, Fukushima Medical University School of Medicine, 1 Hikarigaoka, Fukushima, 960–1295 Japan

## Abstract

Nedd4 is a family of ubiquitin E3 ligases that regulate numerous cellular processes. In this report, we showed that alpha- and beta-herpesviruses have membrane proteins that regulate the function of the Nedd4 family members. Although the homology search score was quite low, UL56 of herpes simplex virus type 1 and 2, ORF0 of varicella-zoster virus, UL42 of human cytomegalovirus, and U24 of human herpesvirus 6A, 6B, and 7 all possess at least one PPxY (PY) motif in their cytoplasmic domain, and are able to bind with Itch, a member of the Nedd4 family. These viral proteins altered the localization of Itch and decreased Itch expression in co-expressing cells. In addition, these viral proteins reduced the production of retrovirus vectors through the regulation of the Nedd4 family of proteins. U24, but not the other proteins, effectively reduced CD3ε expression on the T cell surface. These viral molecules are thought to contribute to the specific function of each virus through the regulation of Nedd4 family activity.

## Introduction

Ubiquitination, which is a post-translational modification whereby a highly conserved 76-amino acid polypeptide is attached to a protein, is essential for numerous eukaryotic cellular processes, including protein turnover, protein sorting, cell cycle control, and signal transduction^1^. The ubiquitination of proteins is mediated by the sequential enzymatic action of ubiquitin-activating (E1) enzymes, ubiquitin-carrier (E2) proteins, and ubiquitin-protein (E3) ligases^2,3^. E3 ligases play a critical role in selecting specific proteins for ubiquitin conjugation. The human genome is known to have at least 600 putative E3 ligases^4,5^. Consequently, the ubiquitin system is very diverse.

The Nedd4 (neural precursor cell-expressed developmentally down-regulated gene 4) family of ubiquitin E3 ligases is found in eukaryotes from yeast to mammals. Yeast have one Nedd4 protein while humans have nine proteins. All Nedd4 proteins contain an N-terminal C2 domain, multiple WW domains, and a C-terminal catalytic HECT (homologous to E6-AP carboxyl terminus) domain^6,7^. The WW domains of Nedd4 proteins interact with the PPxY sequence (PY motif) and are responsible for substrate recognition^6,8^. Nedd4 proteins ubiquitinate their substrate proteins through their interaction with WW domains and PY motifs. A number of Nedd4-interacting proteins (e.g., Ndfip1 and 2) act as adaptors that regulate Nedd4 protein catalytic activity^9^. Mutations in Ndfip1 lead to severe inflammation of the skin and lungs similar to that observed when Itch, a Nedd4 family member, is mutated^10,11^.

Some viruses hijack the catalytic activity of the Nedd4 family of proteins. For instance, the budding of some retroviruses requires Nedd4, Nedd4L and Itch activities through the L-domain of Gag proteins^12–14^. Herpesviruses also have Nedd4-interacting proteins. Herpesviridae is a large group of well-characterized double-stranded DNA viruses consisting of three subfamilies. Among the human herpesviruses, herpes simplex virus type 1 (HSV-1), HSV-2, and varicella-zoster virus (VZV) are included in the alpha herpesvirus subfamily. Human cytomegalovirus (HCMV), human herpesvirus 6 A (HHV-6A), HHV-6B, and HHV-7 are beta-herpesviruses. Epstein-Barr virus (EBV) and Kaposi’s sarcoma-associated herpesvirus (KSHV) belong to the gamma herpesvirus subfamily^15^. HSV, HCMV, and EBV have Nedd4-binding proteins: UL56, UL42, and LMP2A, respectively. These are membrane proteins that contain a small number of PY motifs that interact with the Nedd4 family proteins^16–18^. HSV UL56 and HCMV UL42, in particular, share a characteristic tail-anchored (TA) structure that possesses a trans-membrane domain (TMD) at its C-terminus^17,19^. As alpha, beta, and gamma herpesviruses have Nedd4-interacting proteins, we speculate that all herpesviruses possess Nedd4-interacting proteins. VZV ORF0, a homolog of HSV UL56, also has a PY motif^20^, while the U24 protein of roseolovirus (HHV-6A, HHV-6B, and HHV-7) has a PY motif and a C-terminal TMD. U24 proteins down-regulate CD3ε and transferrin receptors from the T cell surface through the PY motif^21^. In this report, we show that these proteins interacted with Itch, and acted as adaptor proteins for the modulation of Nedd4 protein activity.

## Results

### The interaction between Nedd4 family proteins and viral proteins

**Figure 1:**
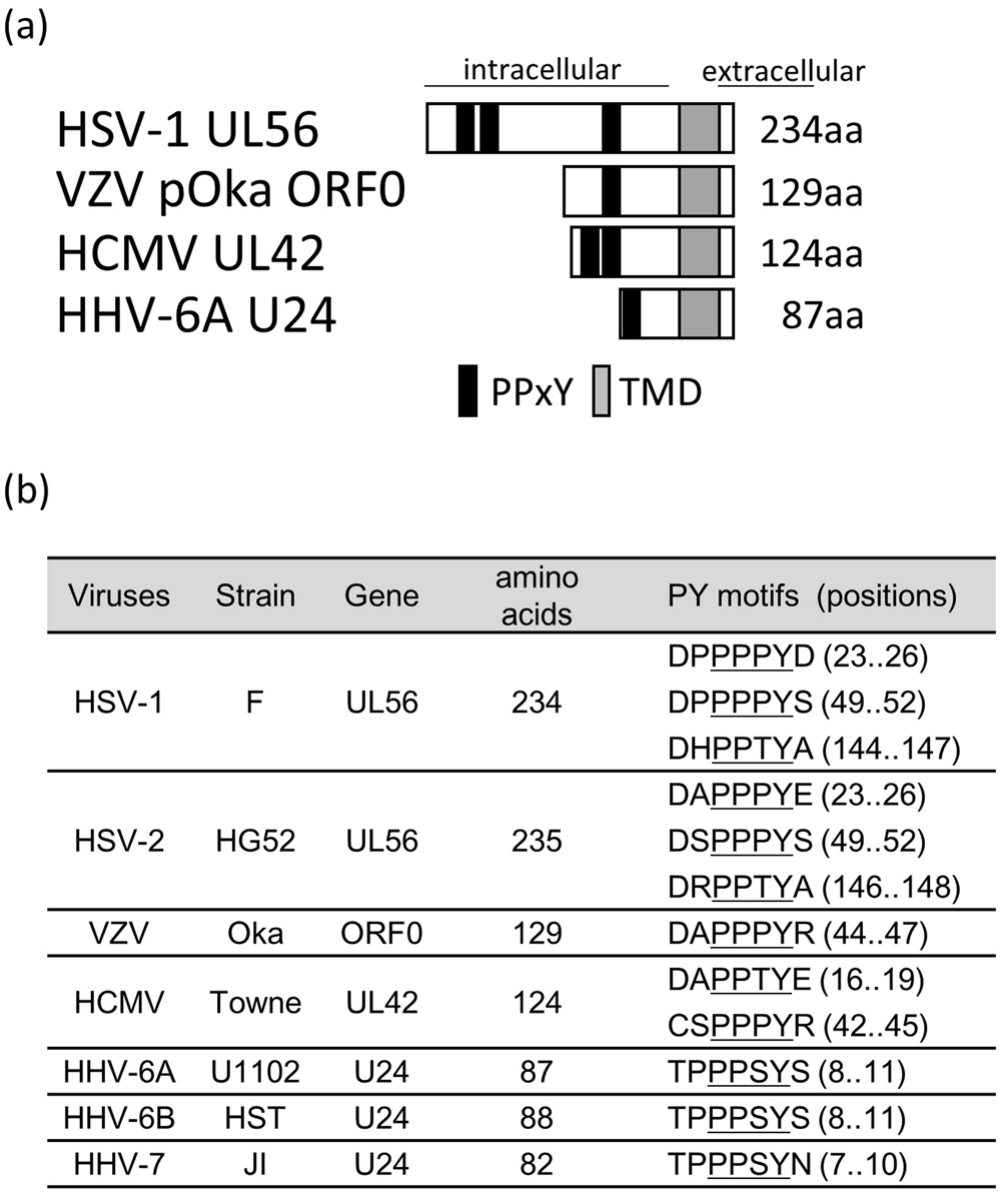
The conservation of viral adaptor proteins for the Nedd4 family among alpha- and beta-herpesviruses. (**a**) Schematic representation of herpesvirus-encoding adaptor proteins for the Nedd4 family used in this study. Rectangles indicate the sizes of the individual proteins. Predicted amino acid numbers are indicated. PY motifs (PPxY) and the transmembrane domain (TMD) are indicated as black and gray boxes, respectively. (**b**) Aligned PY motifs of viral adaptor proteins. Gene name, amino-acid number, the sequence around PY motif and amino-acid positions of PPxY sequence are indicated. The core PPxY sequences are underlined.

According to their amino acid sequences, HSV-1, HSV-2 UL56, VZV ORF0, HCMV UL42, and HHV-6A, -6B, and HHV-7 U24 all have PY motifs at their N-terminus and a putative TMD at their C-terminus (Fig. 1). Although these proteins are not very similar (Table 1), they share a characteristic TA structure, as they have no obvious N-terminal signal peptide. In addition, KSHV ORF16 has a C-terminal TMD and an LPxY sequence, which resembles a PY motif. We therefore hypothesized that these herpesviral proteins are able to interact with Nedd4 family proteins.

**Table 1:** Homology search result scores.

	HSV-1 UL56	HSV-2 UL56	VZV ORF0	HCMV UL42	HHV-6A U24	HHV-6B U24	HHV-7 U24
HSV-1 UL56	1116	696	61	40	46	50	40
HSV-2 UL56		1155	58	59	47	43	37
VZV ORF0			652	59	42	41	43
HCMV UL42				639	63	52	50
HHV-6A U24					470	395	97
HHV-6B U24						462	83
HHV-7 U24							411

**Figure 2:**
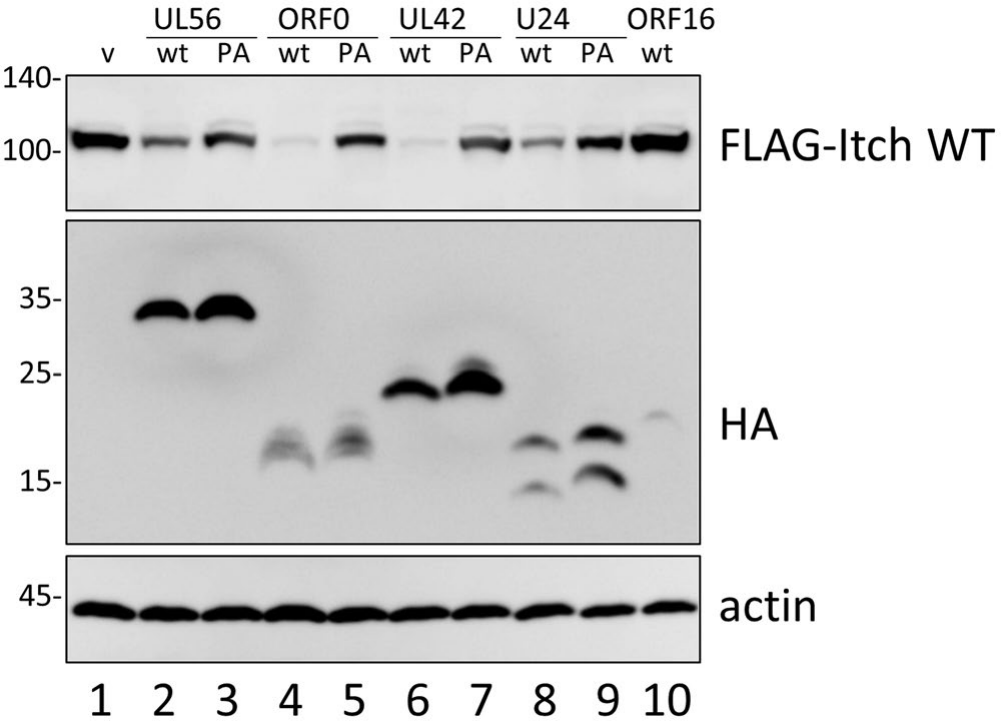
The reduction of FLAG-Itch protein expression in cells co-expressing viral adaptor proteins. HEK293T cells were co-transfected with the FLAG-ItchWT plasmid and HA-tagged viral adaptor proteins, harvested at 24 h post-transfection and analyzed by Western blotting (cropped, full-length blots are given in Supplementary Fig. S1) with the indicated antibodies. Molecular weight mass markers (kDa) are indicated on the left.

To elucidate the effect of herpesvirus proteins on the Nedd4 family proteins, HSV-1 UL56, VZV ORF0, HCMV UL42, and HHV-6A U24, as a representative of roseoloviruses, were co-expressed with wild-type FLAG-tagged Itch (FLAG-ItchWT). The amount of FLAG-Itch was markedly reduced in cells when co-expressed with the wild-type viral proteins but not with PY motif-disrupted (PA) mutants or KSHV ORF16 (Fig. 2). These results suggest that PY motif-containing viral proteins decreased Itch expression.

**Figure 3:**
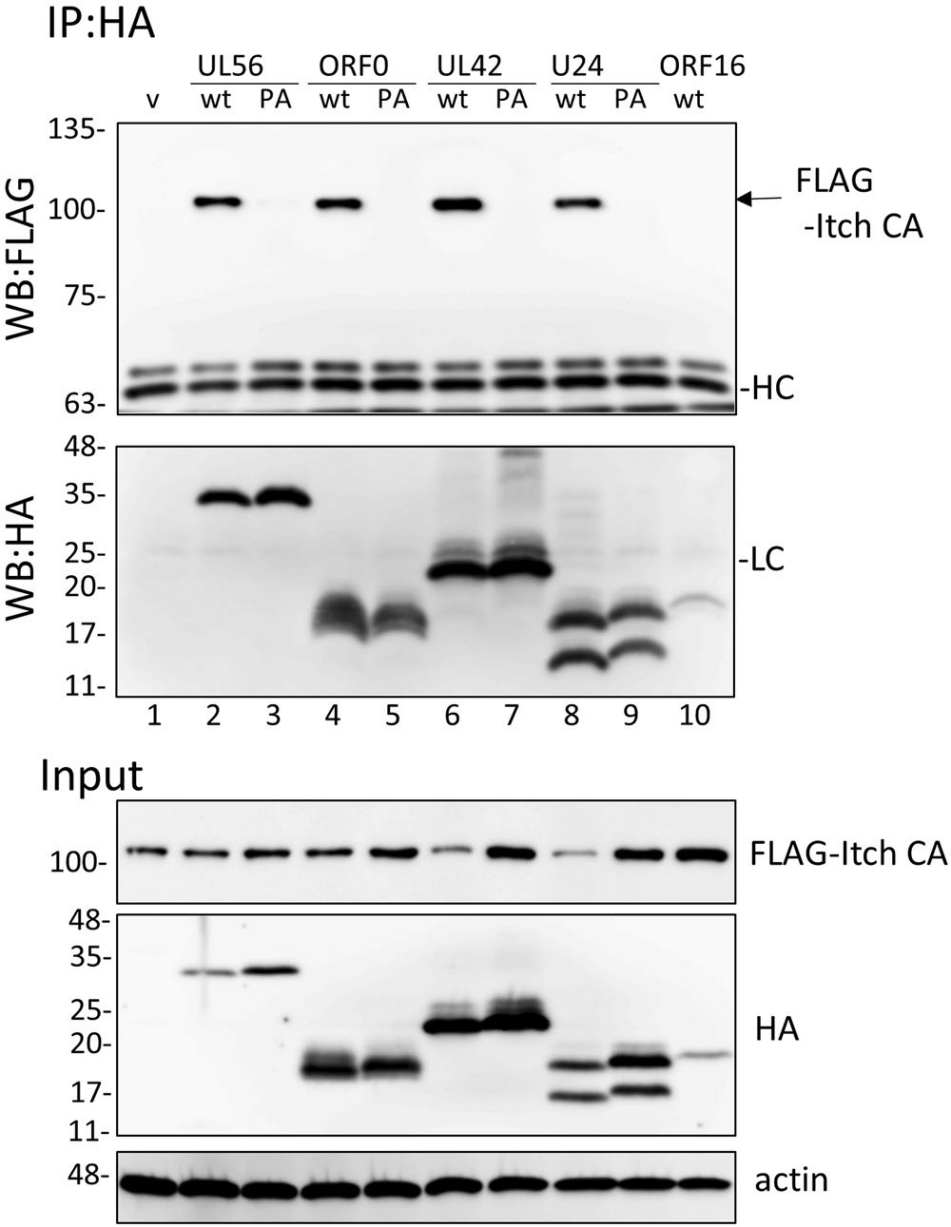
The interaction between Itch and herpesvirus-encoding membrane proteins. HEK293T cells transfected with the plasmids of FLAG-ItchCA and HA-tagged viral adaptor proteins were analyzed by immunoprecipitation assay. Input fractions and immunocomplexes with anti-HA antibody were separated by SDS-PAGE and analyzed by Western blotting with the indicated antibodies (cropped, full-length blots are given in Supplementary Fig. S2). The IgG heavy chain (HC) and light chain (LC) are indicated.

The interaction between herpesviral proteins and Itch was determined by co-immunoprecipitation assay (Fig. 3). FLAG-Itch was expressed in its catalytic negative form (FLAG-Itch CA) in order to prevent intramolecular activation and degradation. As reported previously, FLAG-Itch was co-precipitated with HSV-1 UL56, VZV ORF0, HCMV UL42, and HHV-6A U24 (Fig. 3 lanes 2, 4, 6, and 8, respectively). The interaction was found to be dependent on the PY motifs, as FLAG-Itch was not co-precipitated with PA mutant proteins (Fig. 3 lanes 3, 5, 7, and 9, respectively). In addition, KSHV ORF16 was not co-precipitated with FLAG-Itch (Fig. 3 lane 10).

**Figure 4:**
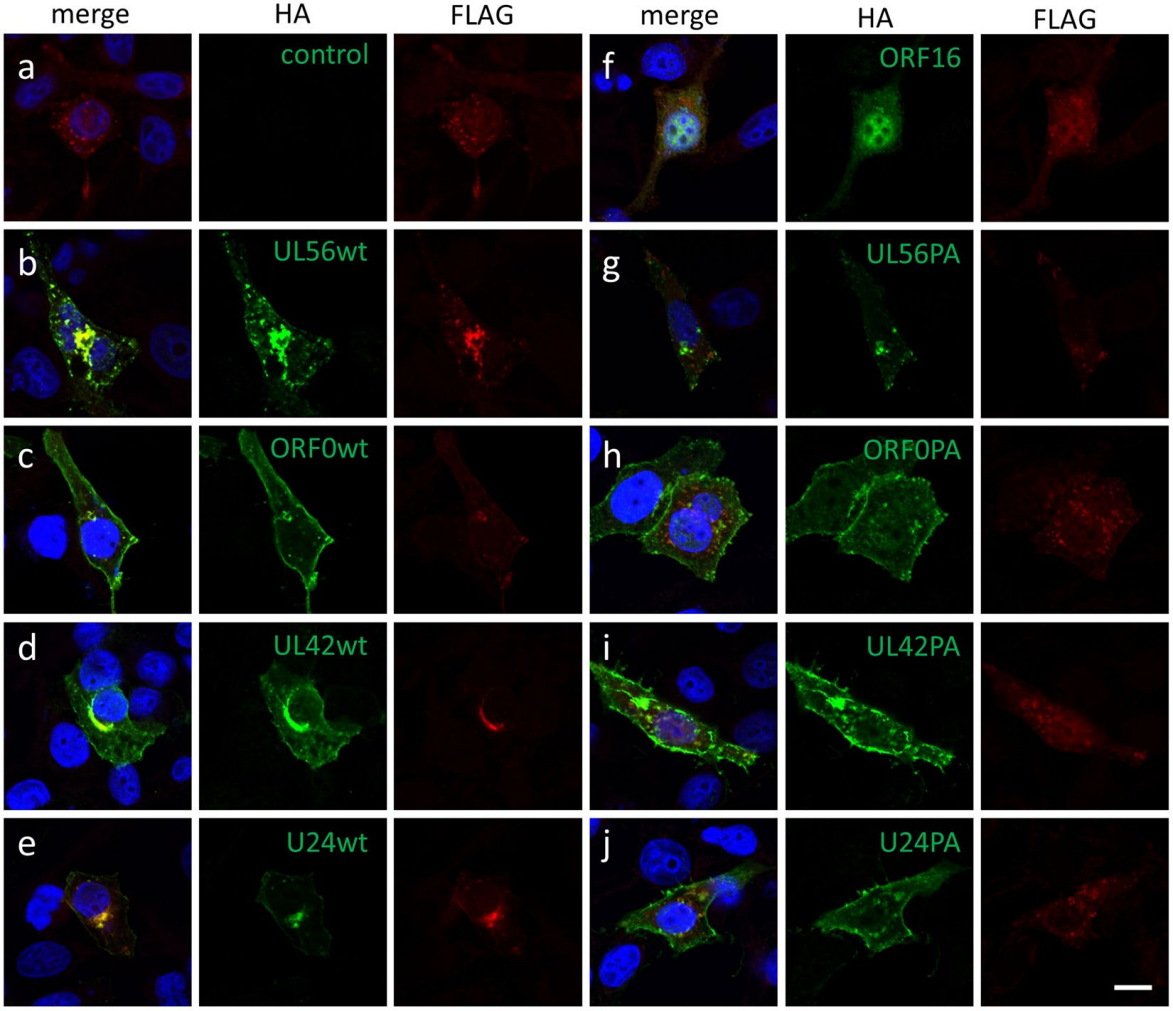
The alteration of Itch subcellular localization by viral adaptor proteins. HeLa cells were co-transfected with the plasmids of FLAG-ItchCA and HA-tagged viral adaptor proteins. Transfected cells were fixed at 24 h post-transfection and analyzed by immunofluorescence assay with the indicated antibodies. The bar indicates 10 µm.

FLAG-Itch proteins were localized to vesicular compartments such as the trans-Golgi network (TGN) and endosomes (Fig. 4a), as described previously^22^. FLAG-Itch proteins were colocalized with the wild-type viral proteins, and accumulated at the perinuclear region of the cytoplasm (Fig. 4b–e). Although PA mutants partially colocalized with FLAG-Itch (Fig. 4g–j), these appeared to be false positives as both Itch and viral proteins are localized to the TGN and endosomal vesicles^17,19,20,22,23^. ORF16 was not able to re-localize FLAG-Itch (Fig. 4f). These data support our co-immunoprecipitation results.

**Figure 5:**
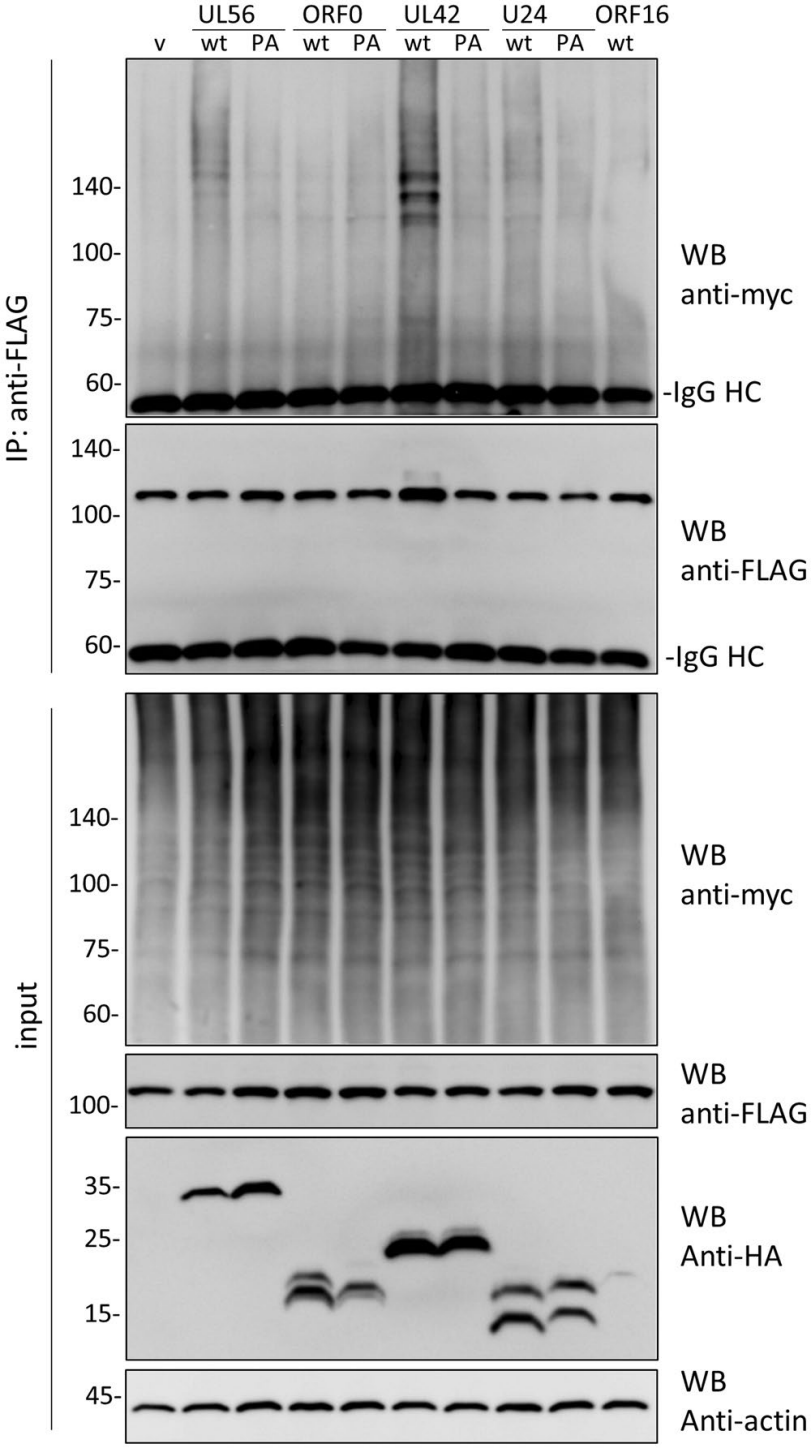
The induction of Itch ubiquitination by viral adaptor proteins. HEK293T cells were co-transfected with the plasmids of FLAG-ItchCA, myc-Ub and HA-tagged viral adaptor proteins in the indicated combinations. After 24 h post-transfection, cells were lysed with RIPA buffer and the lysates were subjected to immunoprecipitation assay with anti-FLAG antibody. Input fractions and precipitated FLAG-Itch CA proteins were analyzed by Western blotting with the indicated antibodies (cropped, full-length blots are given in Supplementary Fig. S3).

As Itch is self-ubiquitinated via an intermolecular mechanism^24^, FLAG-Itch CA was used as a probe for endogenous Itch activation. FLAG-Itch CA, myc-tagged ubiquitin, and herpesvirus proteins were co-transfected and analyzed in an immunoprecipitation assay using an anti-FLAG antibody (Fig. 5). FLAG-Itch CA was highly ubiquitinated in the presence of wild-type HSV-1 UL56 and HCMV UL42, but not in the presence of the PA mutants. In ORF0 or U24 transfected cells, there was no significant difference in the overall levels of FLAG-Itch ubiquitination between the wild-type and PA mutant. These results indicate that these viral proteins have different functions.

### The disruption of retrovirus vector production by viral TA proteins

**Figure 6:**
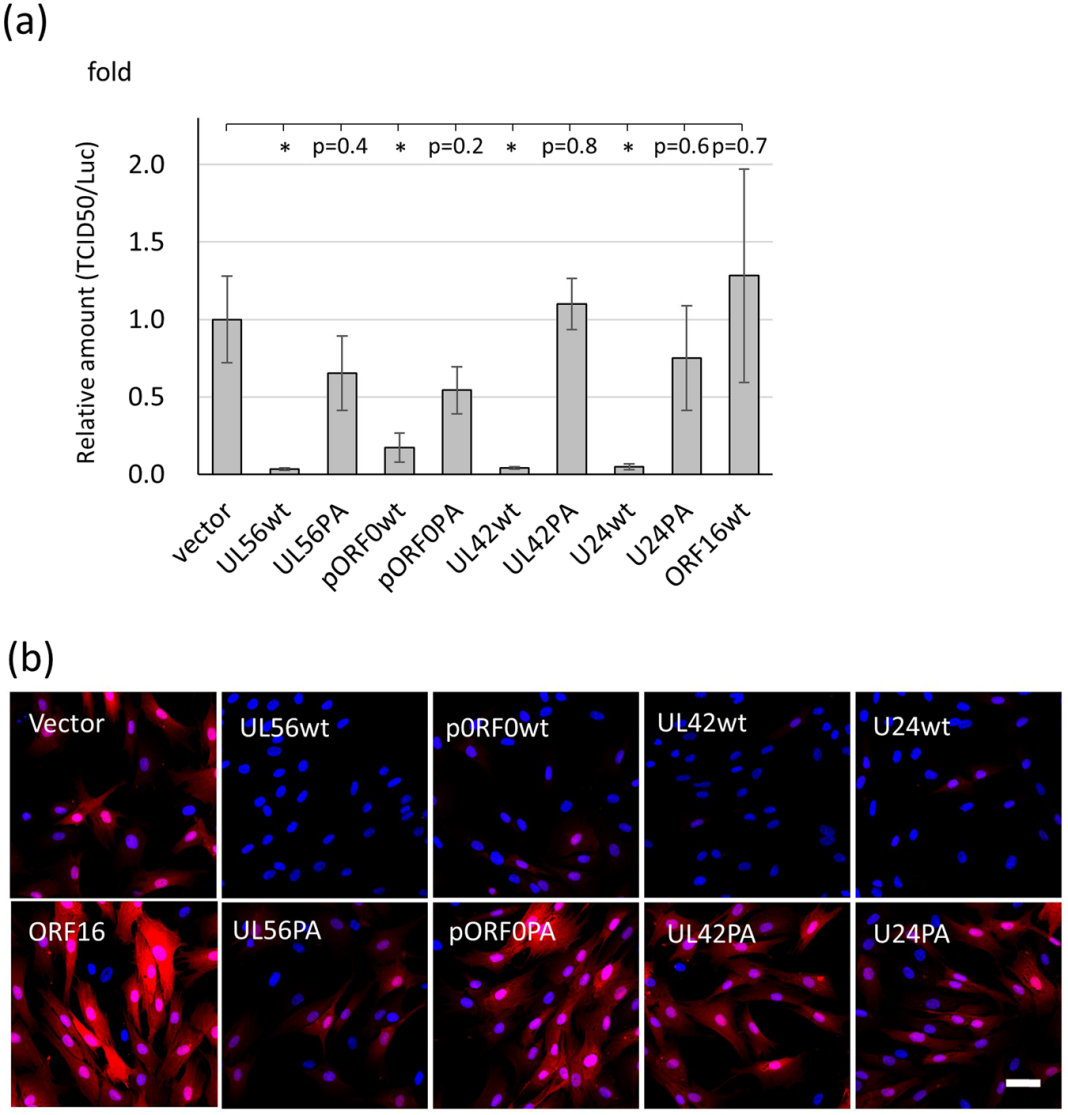
Inhibition of retrovirus vector yield by viral adaptor proteins. (**a**) The packaging cells were co-transfected with mCherry retroviral plasmids and viral adaptor protein-containing plasmids. The retrovirus vector yield was analyzed as the TCID_50_ as described in Materials and Methods. Data are expressed as the mean ± SE of three independent experiments. *P < 0.05 (versus time zero by Student’s t-test). (**b**) The expression of mCherry in retrovirus vector-infected cells was determined in hTERT-BJ1 cells. The bar indicates 50 µm.

The production of retroviral virion-like particles (VLPs) is regulated by Nedd4, Nedd4L, and Itch through Gag ubiquitination when Gag has a PPxY sequence as its L-domain^12^. In order to determine how herpesvirus-encoded proteins modulate Nedd4 activity, we examined the production of retroviral vectors in the presence of herpesvirus proteins. When Nedd4 function was disrupted, the production of retroviral VLPs decreased due to insufficient Gag ubiquitination. The yield of retroviral vectors was measured using the median tissue culture infective dose (TCID_50_) and mCherry fluorescence in susceptible cells as described in the Materials and Methods. The TCID_50_ titer of the retrovirus vectors decreased in the presence of wild-type HSV-1 UL56, VZV ORF0, HCMV UL42, and HHV-6A U24, but not in the presence of the PA mutants or ORF16 (Fig. 6a). The number of mCherry-positive cells was reduced in the presence of herpesviral proteins except for ORF16, and the disruption of PY motifs increased in the mCherry-positive cells (Fig. 6b).

### The down-regulation of CD3ε from a T-cell line

**Figure 7:**
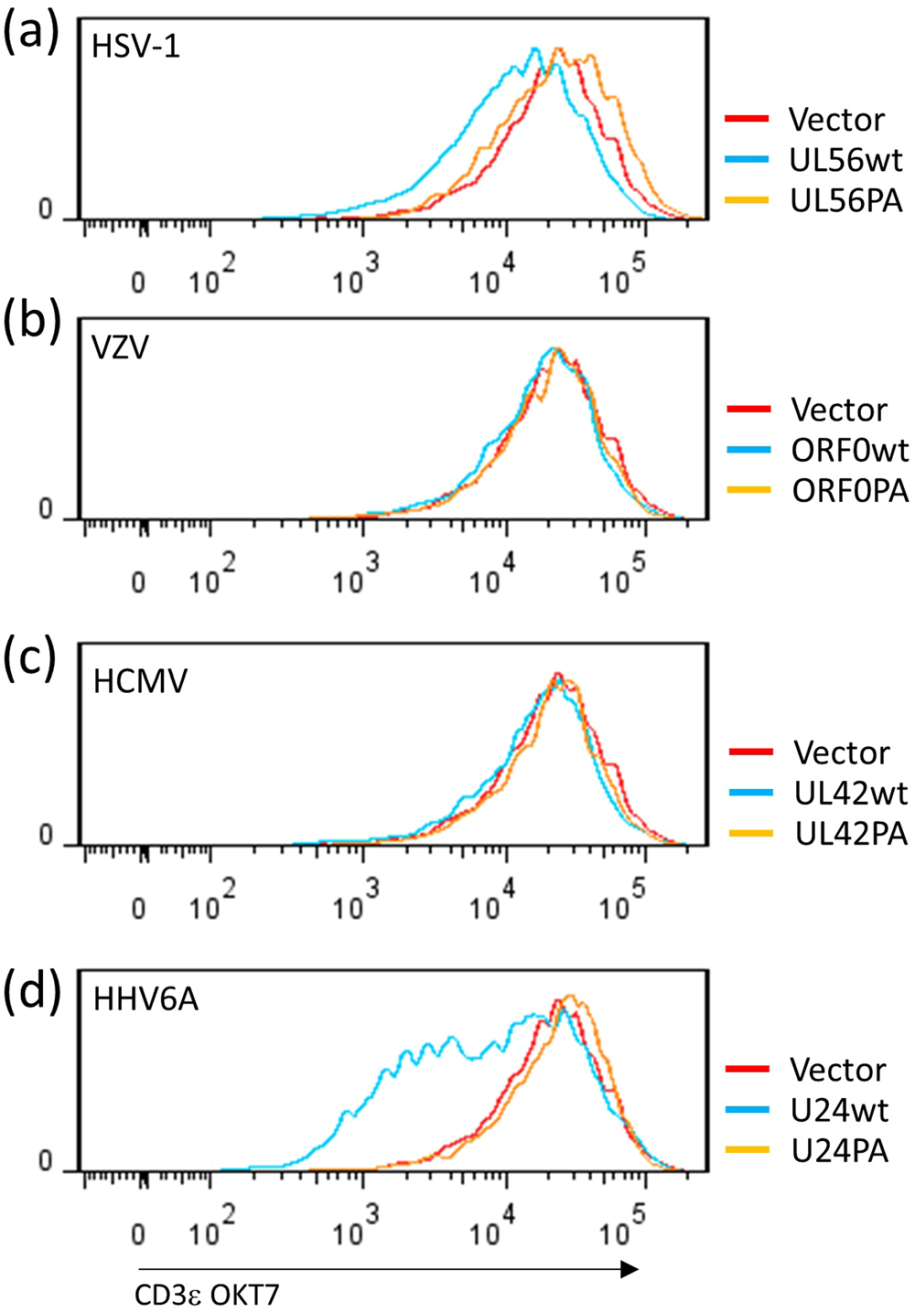
CD3ε down-regulation by herpesvirus-encoding membrane proteins. JM cells were transfected with a bicistronic transcript plasmid encoding herpesvirus proteins (**a**) HSV-1 UL56; (**b**) VZV ORF0; (**c**) HCMV UL42; (**d**) HHV-6A U24 and the PA mutants) and ZsGreen1 (Vector). Twenty-four hours post-transfection, surface CD3ε (OKT7) expression in cells displaying ZsGreen1 expression was analyzed by flow cytometry.

As reported previously, HHV-6A, HHV-6B, and HHV-7 U24 proteins are able to down-regulate CD3ε from Jurkat cells^21,23^. The PY motif of U24 is able to associate with Itch (Fig. 3) and the WW domain of Nedd4^25^. As the PY motif of U24 is responsible for CD3ε down-regulation, we examined whether this function is conserved among all viral proteins. JM cells, a T-cell line, were transfected with pIRES2-ZsGreen plasmids encoding viral proteins, and the surface expression of CD3ε was measured by flow cytometry. HHV-6A U24 down-regulated CD3ε expression through the PY motif (Fig. 7d), whereas CD3ε was only slightly down-regulated by HSV-1 UL56 (Fig. 7a). Further, neither PA UL56 nor any of the other viral proteins tested were able to down-regulate CD3ε (Fig. 7b,c).

## Discussion

We found that HSV-1 UL56, VZV ORF0, HCMV UL42, and HHV-6A U24 were conserved as viral Nedd4 family-interacting proteins. Based on previous reports and sequence similarities, HSV-2 UL56, HHV-6B U24, and HHV-7 U24 can be included in this family^18,21^. Moreover, KSHV ORF16 was not able to interact with Nedd4 proteins, although we suspect that KSHV has Nedd4 family-interacting proteins similar to those observed for EBV. With respect to the aforementioned herpesvirus proteins, we found that they were able to regulate the function of Nedd4 family proteins. Indeed, these viral proteins associated with Itch through their PY motifs to modify Itch function.

Mammalian cells possess certain adaptor proteins for the Nedd4 family, such as Ndfip1 (N4WBP5) and Ndfip2 (N4WBP5A)^26^. These adaptors are able to interact with Nedd4 family proteins through their PY motifs and recruit them to a distinct subcellular compartment^27^. Similarly, viral PY motif-containing proteins interact with Itch and recruit it to a particular cellular location (Figs 3 and 4). As this property is common to cellular Ndfip proteins, these viral proteins should be classified as viral adaptor proteins for the Nedd4 family.

The viral adaptor proteins were found to reduce the amount of wild-type Itch (Fig. 2). However, as Itch CA was decreased to a lesser extent, even in the presence of viral adaptor proteins, E3 ligase activity is thought to be involved in regulating Itch protein expression. As shown in Fig. 5, Itch was highly ubiquitinated in the presence of HSV-1 UL56 or HCMV UL42, but less so in the presence of VZV ORF0 or HHV6A U24. VZV ORF0 and HHV6A U24 might alter the pattern of poly-ubiquitination to decrease the expression or re-localization of the Itch protein. The difference in ubiquitination level might reflect the number of PY motifs possessed by each protein. As shown in Fig. 1a, HSV UL56 and HCMV UL42 have two or three PY motifs. On the other hand, VZV ORF0 and HHV U24 have only one each. Each PY motif in UL56 and UL42 is expected to bind to the identical Nedd4 family proteins, thus multiple E3 ligases would come into close contact on the viral adaptor proteins and ubiquitinate each other.

The ubiquitination of the Gag protein is critical for VLP formation^12,28,29^. Nedd4 family ubiquitin E3 ligase activity is responsible for Gag ubiquitination and VLP formation in retroviruses with a PPxY sequence as an L-domain. As shown in Fig. 6, the retrovirus vector yield was markedly reduced when co-expressed with viral adaptor proteins in packaging cells. Our results indicated that herpesviral adaptor proteins induce a reduction in Nedd4 family protein expression through their interactions. The Nedd4 protein activities would be reduced by these alterations, and subsequently lead to an impairment in VLP formation.

As reported by Sullivan and Coscoy, the PY motif of U24 is responsible for the down-regulation of CD3ε expression on the T cell surface^21,23^. U24 was also found to down-regulate transferrin receptor expression^21^. Based on our results and the report by Sang *et al*., the PY motif of U24 appears to be necessary for interactions between Itch and Nedd4 family proteins. Sang *et al*. reported that the PY motifs of U24 proteins are able to interact with the WW domains of Nedd4 proteins in an acellular system^25^. In addition, the m42 gene product of mouse cytomegalovirus (MCMV), a homolog of HCMV UL42, down-regulates CD45 expression on the MCMV-infected macrophage surface and induces the lysosomal degradation of CD45^30^. The PY motif of m42 is responsible for the degradation of CD45. These findings strongly suggest the involvement of Nedd4 family proteins, or other WW domain containing proteins, in the down-regulation of cell surface protein expression by viral adaptor proteins. Although the possession of PY motifs and interaction with Itch are shared by viral adaptor proteins, only U24 effectively down-regulated CD3ε (Fig. 7d). In the present study, there was no obvious difference between U24 and other viral proteins in terms of Itch binding or its ubiquitination. The binding pattern of Nedd4 family proteins with U24 might play an important role in the down-regulation of CD3ε. As Sang *et al*. described, U24 binds more strongly to Nedd4L WW domains than to Smurf2 WW domains^31^. On the other hand, it is possible that other unknown factors interact with U24 to regulate the activity of Nedd4 family proteins.

In conclusion, the data presented in this study indicate that alpha- and beta-herpesvirus possess conserved Nedd4-interacting proteins as members of the small membrane protein family. Although they were able to bind with Itch, there are some functional differences between these conserved proteins as only U24 was able to regulate CD3ε. Furthermore, the low homology score suggests some functional diversity between these proteins. As VZV ORF0 is a candidate gene for the attenuation of VZV^20,32^, it is important to evaluate the functional differences between these molecules. These distinctive features might be used in the future to determine the properties of these viruses.

## Materials and Methods

### Cells

HEK293T, HeLa and JM (acute T-cell leukemia) cells were obtained from Riken Cell Bank (Tsukuba, Japan). HEK293T, HeLa and retrovirus-packaging Phoenix cells, (Nolan Laboratory, Stanford University, California) were cultured in Dulbecco’s modified Eagle’s medium (DMEM) supplemented with 10% fetal calf serum (FCS). JM cells were maintained in RPMI 1640 with 10% FCS.

### Plasmids

The herpesvirus-encoded genes, UL56 of HSV-1 strain F (UL56), U24 of HHV-6A strain HST (U24) and ORF16 of KSHV strain JSC-1 (ORF16), were amplified by PCR with specific primer pairs. After the first PCR, amplified ORFs were applied to the second PCR with a universal primer, HA-Fw + Koz NotI, and specific reverse primers, respectively. ORF0 of VZV strain pOka (ORF0) and UL42 of HCMV strain Towne (UL42) were amplified from previously reported plasmids^17,20^ with HA-Fw + Koz NotI and specific revisers primers, respectively. The primers used in this study are listed in Supplemental Table 1. The HA-tagged ORFs were cloned into the pIRES-EGFP plasmid (Takara Bio, Shiga, Japan). The PPxY (PY) motifs of these genes were substituted to PPxA (PA) by PCR-based mutagenesis. UL42 PY motif-disrupted mutants (UL42PA) were amplified from the previously reported plasmids^17^ and cloned into the pIRES-EGFP vector. These ORFs without a HA tag were amplified by PCR and cloned into the pIRES2-ZsGreen plasmid (Takara Bio). The FLAG-tagged Itch wild type (ItchWT) and dominant negative form (ItchCA) expression plasmids^22^ were kindly provided from Dr. Angers (Univ. de Montreal). The Myc-tagged ubiquitin expressing plasmid, pCIneo-mycUb, was kindly provided by Dr. Fujimuro (Kyoto Pharmacological Univ.). All plasmids were sequenced.

### Antibodies

The monoclonal antibody against β-actin was provided by Sigma-Aldrich (St. Louis, MO). Anti-HA monoclonal and polyclonal, anti-myc monoclonal and anti-DDDDK tag (FLAG tag) antibodies were provided by MBL (Nagoya, Japan). Anti-mouse IgG Alexa 546 and anti-rabbit IgG Alexa 488 conjugated antibodies were provided by Thermo Fischer Scientific (Waltham, MA)

### Immunoprecipitation

In order to detect protein-protein interactions, transfected 293 T cells were lysed with PBS containing 1% Triton X-100 and protease inhibitor cocktail (Sigma-Aldrich). In order to detect protein ubiquitination, cells were lysed with RIPA buffer (10 mM Tris pH7.8, 150 mM NaCl, 1% Triton X-100, 0.5% Sodium deoxycholate, 0.1% SDS, 5 mM EDTA) containing protease inhibitor cocktail. The immunoprecipitation assay was performed as described elsewhere. In brief, cell lysates were homogenized with 20 strokes of pipetting and then chilled on ice for 20 min. After centrifugation at 15,000 × g for 5 min, the supernatants were clarified by ultra-centrifugation (100,000 × g for 1 h at 4 °C, OptimaMAX-XP, Beckman Coulter, Brea, CA.). The supernatants were precleared with Protein-G Sepharose 4B (GE Healthcare, Little Chalfont, England) for 1 h at 4 °C. After brief centrifugation, the supernatants were immunoprecipitated with specific antibodies binding Protein-G Sepharose 4B overnight at 4 °C. Immunocomplexes were washed 6 times with buffer and analyzed by Western blotting.

### Immunofluorescence assay

Transfected HeLa cells were grown on coverslips. Cells were fixed with cold Acetone-Methanol and subjected to an immunofluorescence assay as described elsewhere^17^. Samples were analyzed by confocal microscopy (FD-1000 Olympus, Tokyo, Japan)

### Preparation and analysis of retrovirus vectors

Phoenix packaging cells^33^ were transfected with pCL-ampho, pCX4bsr-mCherry, and pIRES-EGFP plasmids using Screenfect A reagent (Wako Pure Chemical Industries, Osaka, Japan). PGV-C (Toyo Inc., Tokyo, Japan), a firefly luciferase plasmid, was used as an internal control for transfection. Three days after transfection, viral supernatants were collected and clarified through 0.45 µm filters (Pall. NY). Clarified supernatants were infected to fibroblasts for the analysis of retrovirus vector production. After 3 days post-infection, retrovirus vector-infected fibroblasts were fixed with 4% paraformaldehyde and analyzed using an Olympus FD1000 confocal microscopy system. The retrovirus titer was measured as the TCID_50_ (median tissue culture infective dose), which was divided by the value for firefly luciferase activity.

### Flow Cytometry

In order to transfect JM cells, 5 × 10^6^ cells were mixed with 40ug of the appropriate pIRES2-ZsGreen plasmids and electroporated using a Bio-Rad Gene Purser at 975 uF and 280 V with a Capacitance Extender. Electrophoresed cells were resuspended in RPMI containing 20% FCS without antibiotics and incubated overnight, stained with APC-conjugated anti-CD3 (OKT7) (BioLegend, San Diego, CA) and analyzed using a FACS Canto system (BD Bioscience). The populations of ZsGreen and APC double-positive cells were measured by histogram.

### Homology analysis

The homology score of each pair was determined with Genetyx software version 13 (Genetyx, Tokyo, Japan). Gene accession numbers of the polypeptides were as follows: HSV-1 strain F UL56: AJE60287; HSV-2 strain HG52 UL56: YP_009137209; VZV parental Oka ORF0: YP_053044.1; HCMV strain Towne UL42: ACS32356; HHV6A strain U1102 U24: CAA58404; HHV6B strain HST U24: BAA78245; and HHV7 strain JI U24: AAC54685.

## Electronic supplementary material

Supplementary Information
