# Supplementary material for: Herpesviruses possess conserved proteins for interaction with Nedd4 family ubiquitin E3 ligases
Source: Sci Rep. 2018 Mar 13;8:4447. doi: 10.1038/s41598-018-22682-2 (PMC5849660; doi:10.1038/s41598-018-22682-2)
Supplement: Supplementary file 1 — Supplementary Information [file 41598_2018_22682_MOESM1_ESM.pdf]

Supplementary Information

Herpesviruses possess conserved proteins for interaction with Nedd4 family ubiquitin E3 ligases.

by

Tetsuo Koshizuka, Takahiro Kobayashi, Ken Ishioka, Tatsuo Suzutani.

Supplementary Table S1
The primers used in this study.

Primer name	Sequence (5’->3’)
HSV-1 HAUL56fw	atgtacccatacgatgttccagattacgctgcttcgg aggcggcg
HSV-1 UL56rvEcoRI	ggGAATTCttaccgccacaggaatac
VZV pORF0rvEcoRI	ggGAATTCtcatgtagttgagttggg
HCMV UL42rvEcoRI	ggGAATTCTtatcccgatgttgacac
HHV6A HAU24fw	atgtacccatacgatgttccagattacgctgatcccc ctcggacg
HHV6A U24rvEcoRI	ggGAATTCtcatcgcctttgacgatt
KSHV HAORF16fw	atgtacccatacgatgttccagattacgctgacgagg acgttttg
KSHV ORF16rvEcoRI	ggGAATTCttatctcctgctcatcgc
HA-Fw+koz NotI	gggggGCGGCCGCcATGTACCCATACGATG
HSV1 UL56PA1f	ccgccccccgccgatagcttgtctggt
HSV1 UL56PA1r	caagctatcggcggggggcgggggatc
HSV1 UL56PA2f	cctccaccggcctctgctgggcccctg
HSV1 UL56PA2r	cccagcagaggccggtggaggtgggtc
HSV1 UL56PA3f	cccccaaccgctgccactgtcgtggcc
HSV1 UL56PA3r	gacagtggcagcggttggggggtggtc
VZV ORF0PAf	cagaagccgcgagagtctggtgttc
VZV ORF0PAr	gcaggggggggcgcgtctgccacgg
HHV6A U24PAf	ttcagaggtgctgatgatggacgtcatg
HHV6A U24PAr	gcggacggcggcggcgtccgagg
HSV-1 UL56fw NheI	ggGCTAGCatggcttcggaggcggcg
VZV ORF0fw NheI	ggGCTAGCatggcgaccgtgcactac
HCMV UL42fw NheI	ggGCTAGCATGgagcccacgccgatg
HHV6 U24fw NheI	ggGCTAGCatggatccccctcggacg
KSHV ORF16fw NheI	ggGCTAGCatggacgaggacgttttg

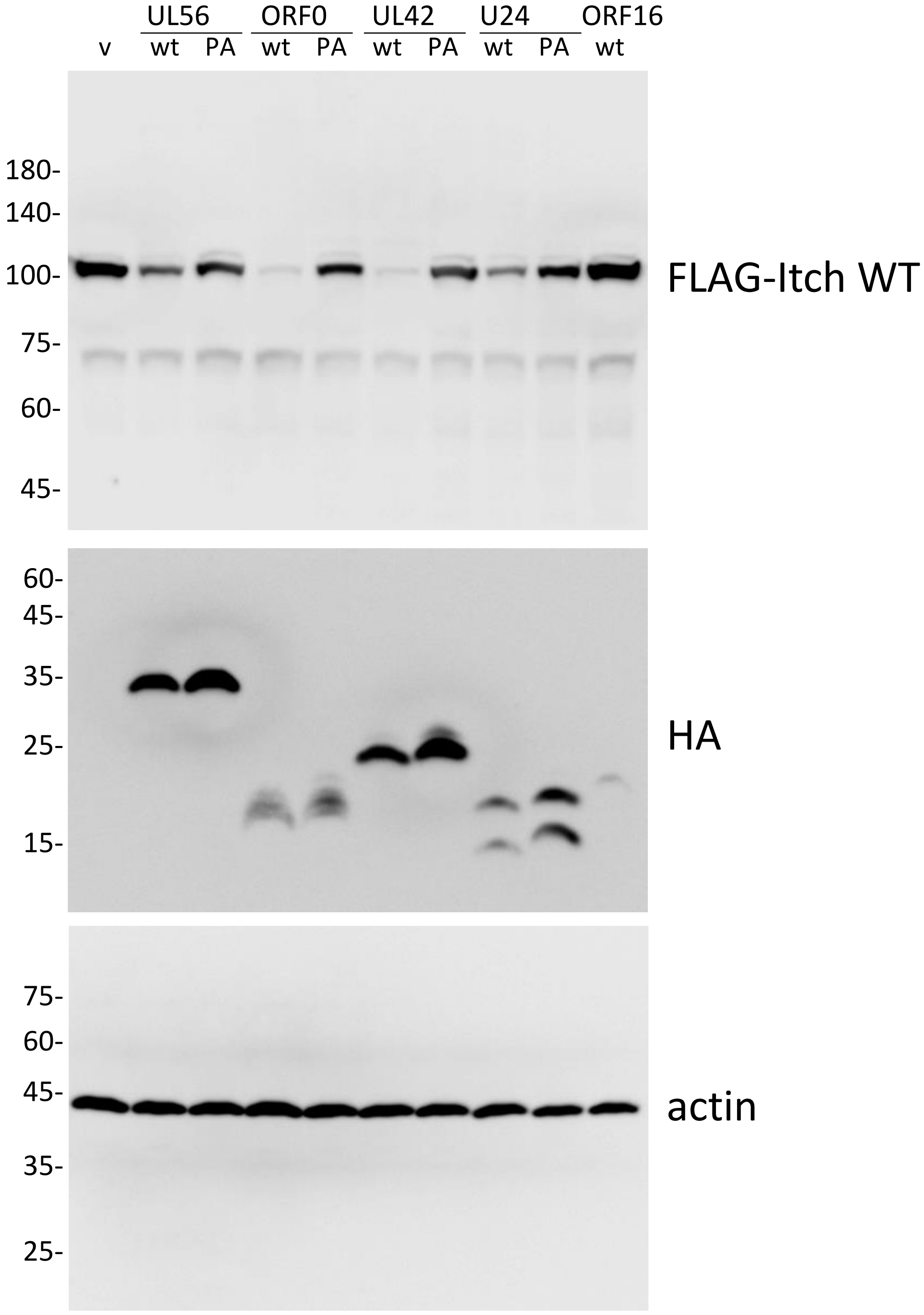

Figure S1. The full-length blot images of Figure 2.

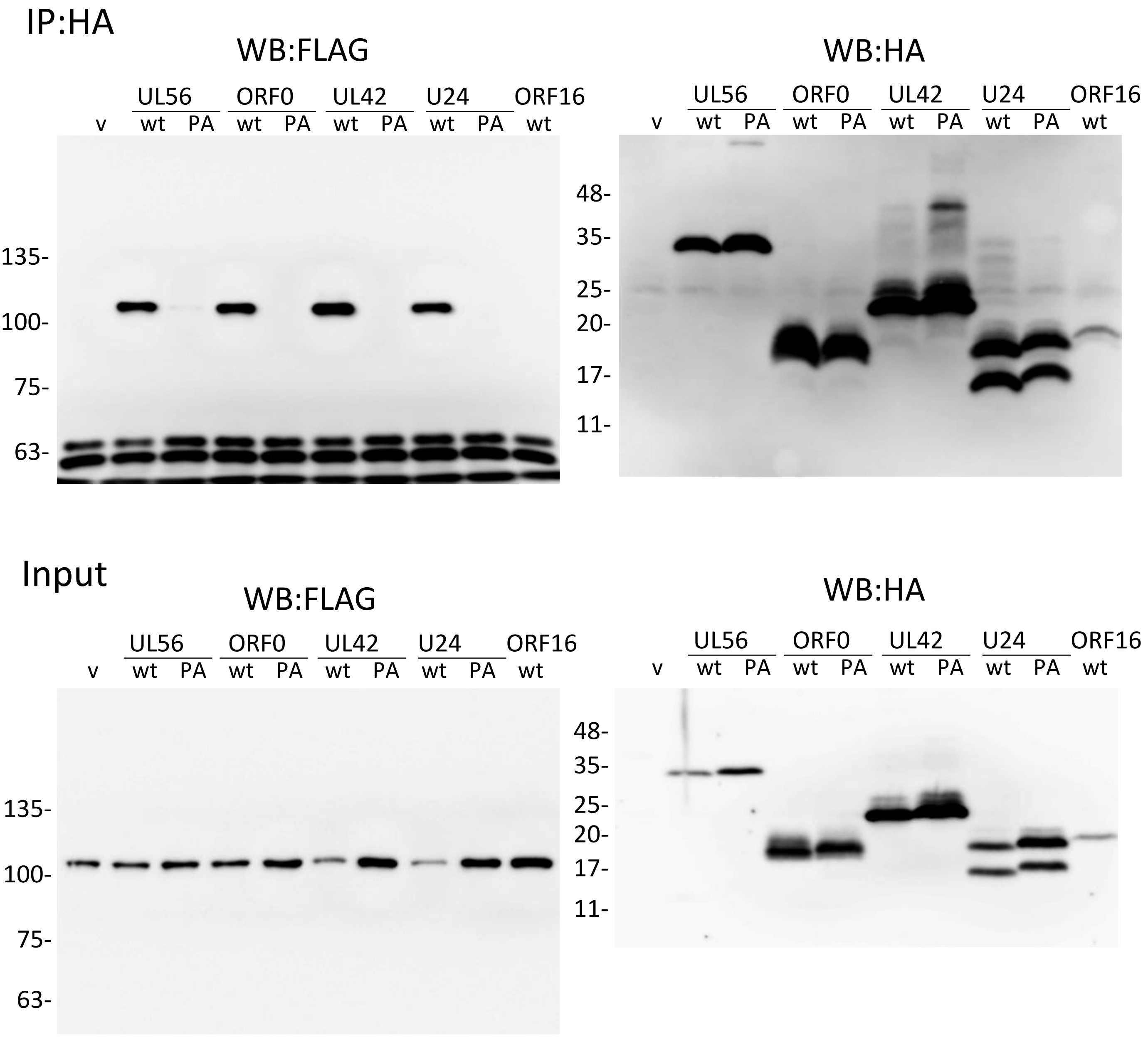

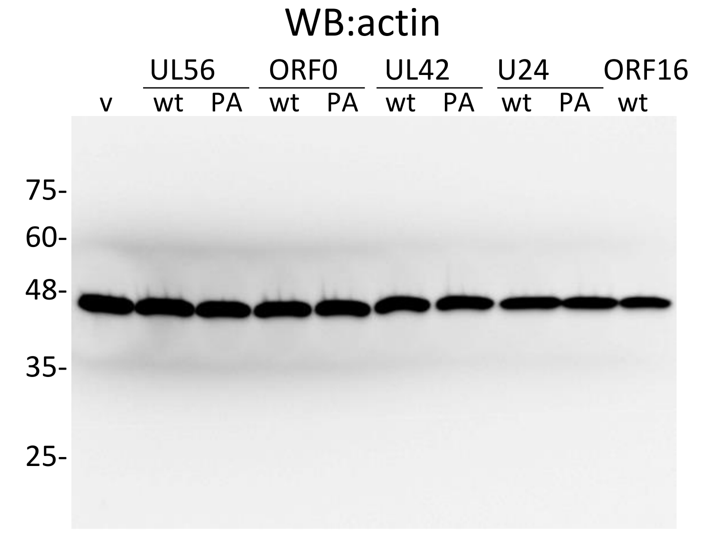

Figure S2. The full-length blot images of Figure 3.

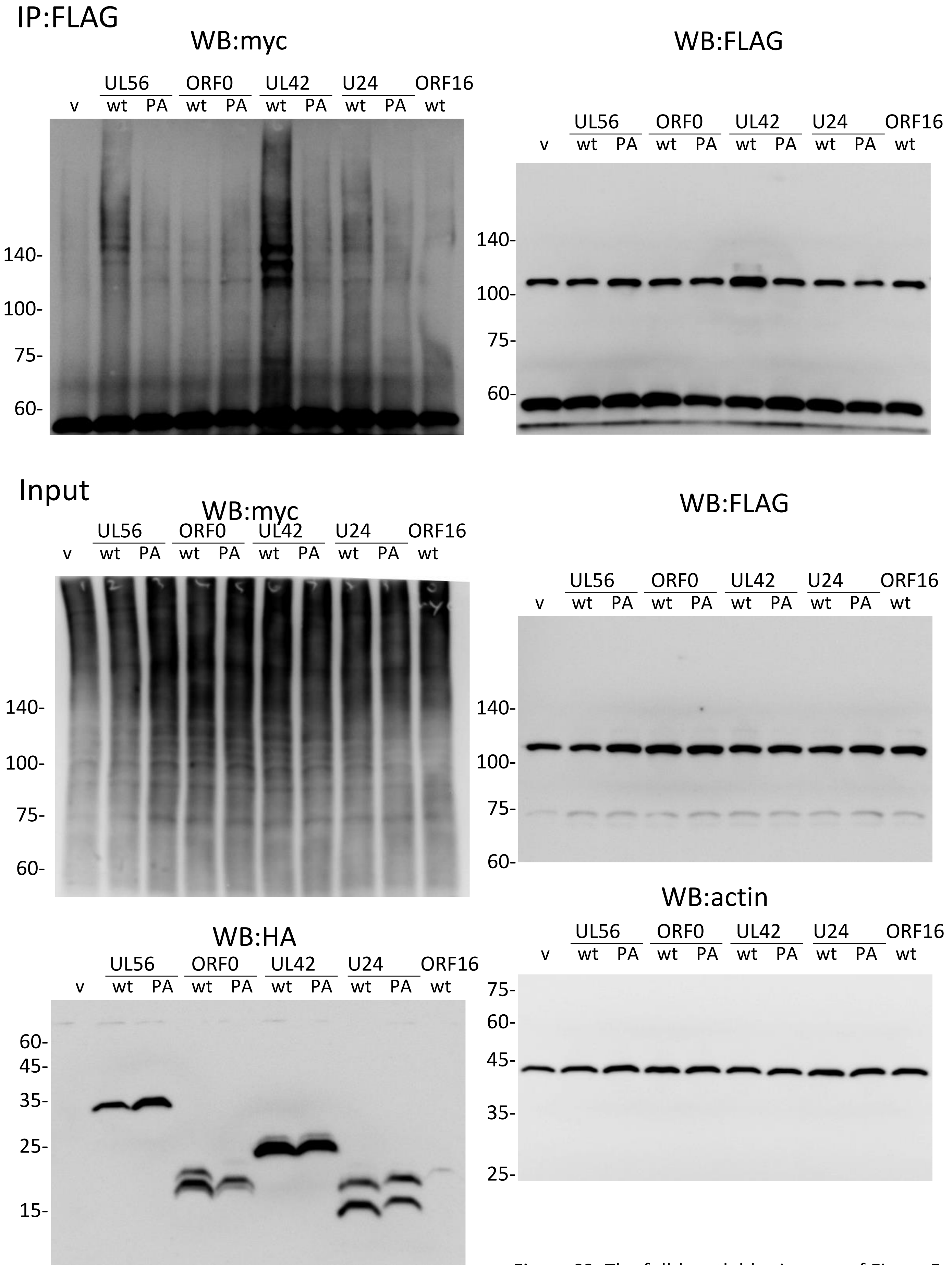

Figure S3. The full-length blot images of Figure 5.
